# Effect of Farming System (Organic vs. Conventional) and Season on Composition and Fatty Acid Profile of Bovine, Caprine and Ovine Milk and Retail Halloumi Cheese Produced in Cyprus

**DOI:** 10.3390/foods10051016

**Published:** 2021-05-06

**Authors:** Ouranios Tzamaloukas, Marina C. Neofytou, Panagiotis E. Simitzis, Despoina Miltiadou

**Affiliations:** 1 Department of Agricultural Sciences Biotechnology and Food Science, Cyprus University of Technology, Limassol 3036, Cyprus; maneofyt@gmail.com (M.C.N.); despoina.miltiadou@cut.ac.cy (D.M.); 2 Laboratory of Animal Breeding and Husbandry, Department of Animal Science, Agricultural University of Athens, Iera Odos, 11855 Athens, Greece; pansimitzis@aua.gr

**Keywords:** organic milk production, cows, goats, sheep, fatty acids, Halloumi cheese, season

## Abstract

The present work aimed to evaluate the effect of farming practices and season on the fat and protein content and fatty acid (FA) profile of milk and Halloumi cheese produced in Cyprus. Over a year, raw bulk-tank milk samples from cow, goat, and sheep farms were collected seasonally from all organic (11) and representative conventional (44) dairy farms, whereas Fresh Halloumi cheese samples were collected monthly from retail outlets (48 organic and 48 conventional samples in total). The different farming practices did not affect the milk fat content of ruminants, while protein levels were decreased in organic bovine and caprine milk. Under organic farming practices, milk and cheese contained increased values of total mono-unsaturated FA (MUFA) and poly-unsaturated FA (PUFA), and specific FA, such as oleic, conjugated linoleic, linoleic, and α-linolenic acids. Total saturated FA (SFA) levels were particularly decreased in organic samples and, consequently, the atherogenic indices of milk and cheese were decreased. Season influenced milk and Halloumi cheese FA profile; spring samples had lower SFA and higher PUFA and MUFA concentrations. Overall, the organic farm practices improved the lipid profile of milk and Halloumi cheese, which is more likely attributed to the different feeding strategies applied in organic dairy farms.

## 1. Introduction

Milk fatty acid (FA) composition can fluctuate widely according to several factors, including animal breed and species, season, stage of lactation, management, and diet [1], with the latter being the predominant factor affecting milk FA profile [2,3]. Several feeding sources, including pasture, conserved forages, concentrates, and oil supplements, can affect, in different ways, the lipid profile of milk and dairy products. For instance, the increase of grass-based forage intake or of vegetable oil, oilseed supplements, or oil by-products levels is associated with an increase in the content of poly-unsaturated FA (PUFA) and individual unsaturated FA, like oleic (C18:1 cis-9, OA), α-linolenic (C18:3n-3, ALA), and conjugated linoleic (CLA cis-9, trans-11; rumenic acid: RA) acids in the milk fat of cows [4,5,6,7] that are beneficially associated with human health and disease prevention [8,9]. On the contrary, when ruminants fed conserved forages, the concentrations of these FA are decreased, while saturated FA (SFA) are increased in milk fat [4]. As a result, seasonal variation in the milk FA profile is observed due to the utilization of grazing-based diets during the summer and ensiled forage diets during the indoor winter period in dairy systems [4]. Studies in north European farms reported that the milk collected during summer contained higher concentrations of PUFA, including CLA and ALA, when compared with milk that was produced during winter when cows were fed silage-based diets [10,11,12,13,14,15].

The different farming systems used in organic production have been reported to induce a desirable effect on milk FA composition of dairy animals (i.e., more unsaturated lipids that are beneficial for human health and lower saturated fats than conventional milk) [16,17,18]. With regards to studies comparing organic and conventional bovine milk derived from farms or retail outlets in Europe, a significantly higher concentration of PUFA was reported for organic milk [10,12,13,14,19,20]. The majority of these studies also demonstrated a higher proportion of CLA content in organic milk, apart from Ellis et al. [13] and Adler et al. [10], who found no difference in this FA. Nevertheless, increased concentrations of total PUFA and CLA have also been reported in studies evaluating organic milk from small ruminants [15,21,22]. According to the previous literature, it is clear that an overall conclusion between organic and conventional farming regarding product quality is difficult to reach for all areas and countries due to the great variability within the production methods, diets, or breeds that used among different regions, and such comparisons should be carried out in each country/area independently [23]. Recently, we compared the metabolite profile of the lipid fraction of organic and conventional bovine milk collected from dairy farms in Cyprus using Nuclear Magnetic Resonance (NMR) metabolomic analysis, only focusing on a minor fraction of lipid components [24]. To date, the effect of organic farming on the milk FA profile from different species in Cyprus, as well as on FA of the increasingly popular worldwide Halloumi cheese that is manufactured from a mixture of goat, sheep, and cow milk has not yet been examined. Furthermore, the effect of season on fat quality of both milk and Halloumi cheese produced in Cyprus has not been previously evaluated. Therefore, the objective of the present work is to determine both the effect of season and applied farming system (organic vs conventional) on the composition and lipid profile of bovine, caprine, and ovine milk and Halloumi cheese retailed in the Cypriot market. 

## 2. Materials and Methods

### 2.1. Sample Collection and Analysis

During a whole year (2014), bovine, caprine, and ovine milk samples from all organic farms of Cyprus were collected (three, five, and three farms, respectively). Conventional milk samples were also collected from 24 cow, 10 goat, and 10 sheep farms that were considered to be representative of the country’s dairy farming according to their annual milk yield. As a standard sampling practice, raw bulk-milk samples from each farm were seasonally collected throughout the year (one sample per farm and season). Thus, a large set of bulk-milk samples accumulated, as follows: 118 bovine (12 organic and 96 conventional), 60 caprine (20 organic and 40 conventional), and 52 ovine (12 organic and 40 conventional). In Cyprus, caprine, ovine, and bovine milk is mainly derived from Damascus goats, cross-bred Chios ewes, and Holstein Friesian cows, respectively. 

After their collection, the milk samples were transferred in cool opaque boxes to the laboratory into 30 mL sterile, screw-top plastic bottles and then stored at −20 °C until further analysis. Measurements for total fat and protein were performed by the application of combined thermo-optical procedures (Lactostar 3510, Funke Gerber, Berlin, Germany) calibrated previously for protein with the Lowry protein assay and fat with the Gerber method 989.05 [25]. 

Moreover, four organic and four conventional fresh Halloumi cheeses were sampled every month from one specific brand, which is the largest of the island, available in supermarkets and other retail outlets, summing a total of 96 samples (48 organic and 48 conventional samples), which were also stored at −20 °C until further analysis. The chemical composition of fresh Halloumi cheese collected was standardized by the manufacturer following the Protected Designation of Origin (PDO) regulations (% crude protein: 28.5%, % fat: 28.6%) [26]. In Cyprus, fresh Halloumi cheese production is made without the addition of starter cultures, and the coagulant that is used for its production is of non-animal origin (e.g., chymosin). The amount of rennet used is ~1 mL 100 mL^–1^ to provide a firm coagulum in about 60 min. Pasteurised sheep and goats milk the primary component, with cow’s milk used in less quantities [26]. 

Lipids from milk were extracted, as described by Tzamaloukas et al. [27]. Briefly, 1.5-mL aliquots of fresh milk were first centrifuged at 17,800× *g* for 30 min. at 4 °C. The resulting separated fat globules (lipid cake) were removed, placed in new tubes, and then allowed to melt at room temperature for 20 min. The samples were then recentrifuged at 19,500× *g* for 20 min. at room temperature, and 20 mg aliquots of the resulting lipid cake were removed to new tubes and dispersed in 1 mL of n-hexane by shaking. 

The cheese fat was extracted with diethyl ether according to the method described by Neofytou et al. [28]. Briefly, 50 mL of diethyl ether and 0.5 g of Na_2_SO_4_ was added to 30 to 40 g of cheese samples and mixed vigorously. After 15 min., the mixture was filtered through filter paper (Whatman no. 4, Sigma-Aldrich, St. Louis, MO, USA) from a funnel. The filtrate was centrifuged for 2 min. at 2500× *g* at room temperature to remove the undesired particles that originated from cheese. The liquid phase of diethyl ether and oil was taken into a centrifuge test tube and diethyl ether was removed using a rotary evaporator at 40 ± 1 °C. Subsequently, the sample was flushed with nitrogen to remove the remaining ether from oil. 

Fatty acid methyl esters (FAME) of milk and Halloumi cheese lipids were prepared by transesterification with methanolic potassium hydroxide according to the ISO [29] method. The FAME profiles were generated using a GCMS-QP2010 Plus Gas Chromatography-Mass Spectrometer (Shimadzu, Duisburg, Germany) that was equipped with a 50 m × 0.25 mm × 0.2 μm column (Agilent CP-Sil 88 fused- silica capillary column) with a split ratio of 1:20. The column was held for 1 min. at 80 °C after injection, increased at 20 °C/min. to 120 °C, then raised to 193 °C at 1 °C/min., and finally increased to 220 °C at 5 °C/min. Helium was the carrier gas at 1 mL/min., with both injector and inter-face temperatures of 225 °C. Chromatographic profiles were analyzed using Shimadzu GCMS Postrun Solution software (GCMSsolution, Shimadzu, Duisburg, Germany). Individual peaks were identified by comparison of their retention indices and mass spectra to those of commercially available standards (Supelco 37-FAME standard mix, CLA cis-9, trans-11, CLA trans-10, cis-12, C18:1 trans-11; Sigma-Aldrich, Gillingham, UK) and mass spectral libraries (NIST) quantitated by peak integration and expressed as a percentage of the total fat.

### 2.2. Statistical Analysis

All of the data on the composition of milk and Halloumi cheese were analyzed based on mixed-effects models with 2 × 4 factorial treatment design representing the farming system (F) and season (S) as the main fixed effect factors, using the SPSS 20 software (StatSoft Inc., Tulsa, OK, USA). The results are presented as least square means ± standard error of the means (SEM). Because of the lack of significant interactions, the main effects for the season were compared when appropriate using Tukey’s Honestly Significant Difference (HSD) test. Significant differences for both fixed main effects were considered at *p* < 0.05, and *p*-values within 0.05 and 0.10 were regarded as tendencies.

## 3. Results

### 3.1. Effect of Farming System and Season on Milk Composition

The fat percentage was neither affected by the farming system nor by season in the milk produced from all species (Table 1). On the contrary, the protein content was decreased by 4 and 7% in organic bovine (*p* < 0.01) and caprine (*p* < 0.001) milk, respectively, while it was similar in the organic and conventional ovine milk samples. Seasonally, there were no significant differences in the proportion of protein milk of cows and ewes, while the caprine milk samples collected during the winter had a significantly higher (*p* < 0.01) percentage of protein when compared with the other three sampling periods. 

### 3.2. Effect of Farming System and Season on Milk FA Profile

Table 2, Table 3 and Table 4 presents the bovine, caprine, and ovine milk FA composition, respectively. The concentration of individual SFA, such as C12:0, C14:0, and C16:0, were lower (*p* < 0.001), while that of C18:0 was higher in organic as compared with conventional milk samples, with the exception of some FA in ovine milk. For instance, there were no significant differences in C12:0 and C18:0 levels, while a tendency for decreased C14:0 concentration was observed in organic ewe milk. The levels of total MUFA were increased (*p* < 0.001) in organic milk of cows and goats, while, in ovine milk, no statistical differences were observed. Regarding the concentrations of individual 18-C MUFA, such as OA (C18:1 cis-9), they were increased in organic milk of cows (*p* < 0.001), goats (*p* < 0.001), and ewes (*p* < 0.05), while the content of vaccenic acid (C18:1 trans-11, VA) and other C18:1 isomers was only enhanced in organic bovine and ovine milk. In contrast, the contents of other MUFA, i.e., C10:1 cis-9, C12:1 cis-9, C14:1 cis-9, and C16:1 cis-9 acids, were decreased in organic bovine and caprine milk fat, while, in ovine milk, the levels of these FA were not affected by the applied farming practices. 

The content of total PUFA was enhanced between 43 and 67% in bovine, caprine, and ovine milk as a result of the organic farming practices (*p* < 0.001). Additionally, the concentrations of RA and ALA were significantly increased in all types of organic as compared with conventional milk samples (*p* < 0.05). At the same time, the levels of linoleic acid (C18:2n-6, LA) were elevated between 36, 40, and 30% in organic bovine, caprine, and ovine milk. No significant differences due to the applied farming system in the percentage of arachidonic acid (C20:4n-6) were observed. The LA:ALA ratio was significantly decreased in bovine (*p* < 0.05), tended to be decreased in caprine (*p* < 0.1), and was not affected in ovine organic milk. Additionally, the atherogenic index was significantly diminished within 26 and 33% in organic compared with the conventional milk of cows, goats, and ewes. 

A considerable variation in bovine, caprine, and ovine milk FA content was observed due to the season of production (values presented in Table 2, Table 3 and Table 4, respectively). The levels of long-chain SFA and nutritionally less desirable individual SFA, including C14:0 and C16:0, were significantly lower in bovine and caprine milk collected during spring as compared with the other three seasons, while opposite results were observed for the C18:0 content (*p* < 0.05). Concentrations of total PUFA were increased by 13 and 17% in cow and goat milk collected during spring, respectively. The average percentage of RA was higher in the milk of cows and ewes collected during spring when compared with the other seasons, while the LA concentrations were only statistically enhanced in the caprine milk during spring. Additionally, the percentages of VA and other C18:1 were significantly higher in all types of milk collected during spring, while the highest values for the major MUFA, like OA (C18:1 cis-9), were observed in autumn for cows (*p* < 0.001) and in winter for goats (*p* < 0.01). Nevertheless, the milk atherogenic index was decreased during spring, while the LA:ALA ratio did not differ across seasons in all types of milk. 

### 3.3. Effect of Farming System and Season on Halloumi Cheese FA Profile 

Table 5 presents the differences in fresh Halloumi cheese FA composition due to farming system and season of sampling. The results showed differences in retail Halloumi FA composition between organic and conventional cheese; organic Halloumi contained increased levels of 18-C FA, and decreased the total SFA (*p* < 0.001), particularly short- and medium-chain SFA between C6:0 to C14:0. Furthermore, the concentrations of total MUFA and the OA, VA, and other C18:1 isomers were enhanced in retail organic Halloumi cheese (*p* < 0.001). It is also noteworthy that the concentrations of total PUFA were higher by 26% in organic as compared with the conventional cheese (*p* < 0.001), with the average percentages of individual PUFA, namely LA, ALA, and RA, being particularly enhanced, by 25%, 20%, and 28%, respectively. No farming effect in the levels of C20:4n-6 as well as in the LA:ALA ratio of cheese fat was observed. The atherogenic index of Halloumi cheese was also affected and diminished by 15% in the organic samples when compared with the corresponding conventional.

The effect of season on the FA composition of retail Halloumi cheese was similar to that on milk FA composition of cows, goats, and ewes. In particular, the concentrations of total SFA and individual FA, such as C14:0 and C16:0, were lower in the fat of cheese produced in spring as compared with that manufactured during the other three seasons. In accordance, the average percentages of 18-C FA, like VA, were the highest in spring products (*p* < 0.001), while no seasonal effect in OA or other C18:1 isomers of cheese fat was observed (Table 5). The PUFA content of cheese in spring was significantly higher (*p* < 0.001) than the content in all other seasons, showing, on average, a 15% increase. Regarding the results of individual PUFA, the average percentages of LA, ALA, and RA in Halloumi cheese produced during spring was significantly increased when compared with that produced during the other seasons. There was no difference in the cheese LA:ALA ratio and the concentration of arachidonic acid across seasons. Finally, the atherogenic index was significantly lower in Halloumi cheese that was collected during spring compared with the other seasons.

## 4. Discussion

### 4.1. Effect of Farming System on Milk Composition and FA Profile

In the present study, the milk fat content was not affected by the organic farming practices in all ruminant species. This is consistent with previous studies examining organic and conventional milk composition of cows [10,11,30,31], goats and ewes [21,32], although there are researchers that found an increased [12] or a decreased fat content in organic milk from cows [33] or small ruminants [15,22,34]. These contradictory reports may be attributed to different diets that are offered or breeds used or several factors that can influence milk fat composition in such comparisons [23]. Nevertheless, the protein content of milk in the present study was affected by farming practices due to higher grazing/forage consumption showing significantly decreased values for bovine and caprine organic milk and, numerically, but not significantly, lower values in organic ovine milk. These results have been observed in previous reports indicating a decreased protein content in the organic milk of cows [11] and goats [32], while other studies showed no effect in organic ovine milk [22,34]. Schwendel et al. [23], who reviewed several studies in organic and non-organic dairy cattle farms, reported a tendency for conventional milk to contain a higher concentration of protein, and this was attributed to starch-based supplements, which are commonly used in higher amounts in conventional diets and increase both energy intake and milk protein content. Although detailed feeding rations were not possible to be collected in the context of the present work, this reason could explain the decreased organic milk protein levels that are shown in the present study, since the conventional farms in Cyprus use extremely high amounts of concentrates (up 60 to 70% of intake) as opposed to forage-based organic diets.

The results showed that organic farming practices positively affected the milk FA profile of all species’ by reducing SFA and increasing individual FA with beneficial effects for human health. Similar results are found in UK dairy farms, since milk that originated from organic production systems has an increased nutritionally desirable PUFA content, i.e., RA, ALA, eicosapentaenoic, and docosapentaenoic acids, and reduced the levels of the undesirable palmitic acid [35]. Forage intake (grazing pasture, hay, and silage) is one of the main differences between organic and conventional farms, since organically reared animals are expected to consume at least 60% of their diet (dry matter) as forage. These leafy pasture plants are rich in n-3 fatty acids and their consumption leads to milk with increased n-3 PUFA content [36]. However, proportions of specific C18 FAs, like LA. are present in even higher proportions in milk from silage-fed (mainly corn silage) cows than in milk from grazed cows, in which the proportion of LA is predominantly low [37]. Cows that were fed grass silage also produced milk with higher C18:0 and trans C18:2 levels when compared with cows provided with corn silage [38]. 

The levels of total PUFA and individual 18-C FAs, such as OA, RA, LA, and ALA, were increased, while the concentration of total SFA, and particularly C14:0 and C16:0, and consequently the atherogenic index, were significantly decreased, as shown in the present study. These results are in agreement with the findings of several studies examining the effect of organic management system on the milk FA profile of cows [6,10,12,13,14,19,20,24,39,40,41] and small ruminants [15,21,22]. Similarly, the meta-analysis studies of Palupi et al. [16] and Srednicka-Tober et al. [17], who summarized the results of 29 and 196 studies in ruminants, respectively, confirmed that organic milk contained more PUFA and n-3 PUFA than conventional milk with concomitant decreased or non-affected values for SFA. This trend has been extensively reported in the literature and, when it is not observed, other mechanisms have been involved. For instance, Adler et al. [10] in Norway reported higher concentrations of SFA in organic milk, and this finding was attributed to the negative energy status of the cows when milk samples were collected that caused an alteration in the milk FA profile. 

The concentrations of total MUFA were significantly increased in organic bovine and caprine milk, and these results are in agreement with the findings of Butler et al. [39], who performed a controlled experiment in cows and with several studies in small ruminants [15,22]. Nonetheless, other studies also reported a decrease [10,13,19,40] or no significant effect [11,12,14,30,31] in the content of MUFA in organic cow milk. Likewise, the two meta-analysis studies of Palupi et al. [16] and Srednicka-Tober et al. [17] demonstrated contradictory results regarding MUFA milk content. The former detected significantly decreased MUFA concentrations in organic milk, while the latter reported no significant effect between organic and conventional farms, indicating that specific feed components in diets may have affected this particular FA group. It is likely that the differences highlighted in the FA profile between ruminant species are mostly due to feeding; for instance, in small ruminants that are traditionally reared in semi-extensive systems, pasture feeding may have removed the differences between organic and conventional milk. Pasture grazing is the main parameter that differentiates bovine milk FA profile of organic vs conventional farms, as also indicated by Scwendel et al. [42].

### 4.2. Effect of Season on Milk Composition and FA Profile

Season did not affect the fat percentage of cow, goat, and ewe milk in the present study. However, the reduction of fat and protein content is consistently reported by farmers and the dairy industry in Cyprus (personal communications) due to heat stress during the summer months. Nonetheless, this was not observed in the present study, since fat and protein content was similar across seasons in all species, with the only exception of protein content of goats, which was found to be elevated in milk that was collected during winter. Increased milk protein content has been reported in the winter season, in temperate climates, when the concentrate-to-forage ratio of diets is higher as compared with grazing season (spring, summer), and this has been associated with higher propionic acid production in the rumen and increased microbial protein supply [43]. Nevertheless, studies in ruminants show controversial results on the effect of season on milk protein content reporting no effect [10,12], a significant decrease in summer milk [31], or an increase in winter milk [15].

The content of total PUFA, MUFA, including RA and VA, was higher, while the SFA concentrations were lower in milk that was collected from all species during spring when compared with other seasons. Similar effects have been reported in the milk that was collected during summer months from cows [10,12,13,31,41,44] and small ruminants [15,45] as compared with winter sampling. Higher LA and omega-3 levels in ovine milk and the derived cheese were also observed in spring when compared with winter in an experiment implemented in central Italy, a fact that is possibly related with the increased linolenic acid content of the pasture [46]. Similar results were illustrated for caprine milk; a higher omega 3 to omega 6 PUFA ratio was observed in spring than summer in a study originated from the same country [47]. Pasture grazing was also associated with increased bovine milk PUFA and CLA content in low-input mountain farms of Poland [48]. It has been demonstrated that, during the conservation processes of forages (wilting and ensiling), especially during winter, extensive lipolysis and oxidative loss of PUFA occurs due to plant metabolic mechanisms [5]. In addition, the grass in pasture is generally less mature than the grass that is cut for hay or silage manufacture, leading to decreased levels of PUFA, mainly C18:3 in conserved grass compared with fresh grass [49]. Therefore, increasing the intake of fresh forage in dairy diets, which is a usual practice during summer in most of the European farms and during spring months in Cyprus leads to elevated levels of MUFA, PUFA, and individual unsaturated fatty acids, like RA and VA, in milk due to a combination of increased dietary supply of PUFA and MUFA and shifts in the rumen biohydrogenation (BH) process or desaturase activity in the mammary gland [5]. 

### 4.3. Effect of Farming System and Season on FA Profile of Halloumi Cheese

Interestingly, the effects of organic farming system on the FA composition of retail Halloumi cheese were similar to those that were observed in the organic bovine, caprine, and ovine milk. More specifically, a reduction in the concentration of SFA and in the atherogenic index was demonstrated similarly to the organic milk FA results of all ruminant species, while increased levels of PUFA and MUFA were observed in organic Halloumi cheese, as compared with conventional ones that were collected throughout the year, a result that is line with the results observed in organic cow and goat milk. Organic Halloumi cheese had increased content of 18-C FA, including OA, VA, RA, LA, and ALA, agreeing with the results of Prandini et al. [50] and Bergamo et al. [51], who reported increased proportions of VA, ALA, and RA in organic Grana Podano and Mozzarella cheeses, respectively. Furthermore, the seasonal variation in FA composition of retail Halloumi cheese was found in the present study, similar to those that were observed in bovine and caprine milk. Fresh Halloumi cheese that was collected in spring contained more total PUFA and MUFA as well as higher concentrations of specific FA, like vaccenic, ALA, and RA, with a concomitant reduction of SFA and cheese atherogenic index when compared with the other three sampling seasons. Those results agree with previous studies [52,53] evaluating the effect of season on the FA composition of cheese reporting an almost similar FA profile in milk and cheese. It has been reported that heating, the fermentation culture used, and the ripening time required for the production of cheese could potentially modulate cheese FA composition [4]. However, in our previous studies [28,54], the FA profiles of milk and related Halloumi cheese produced were similar, which suggested that the improvement in nutritional quality achieved in milk due to organic practices is possibly further maintained in organic Halloumi cheese in the present study. 

## 5. Conclusions

The current work is the first large-scale study undertaken in a semi-arid, Mediterranean type of climate, such as that of Cyprus, investigating the effects of farming system (conventional vs organic) and season on milk composition and lipid quality of milk and Halloumi cheese. The major finding of the present work is related to organic milk and cheese produced. Although similar fat content was observed in organic and conventional milk, the protein percentage was slightly decreased in organic milk. Regarding FA profile, organic milk from all species and organic Halloumi cheese had improved quality, since they contained lower total SFA levels and individual ones, like palmitic acid, while the only elevated SFA was stearic acid compared with conventional milk and cheese. Additionally, higher MUFA, PUFA, and individual FA levels, such as oleic, vaccenic, rumenic, linoleic, and linolenic acids, all related with beneficial effects for human health, were observed in both organic milk and Halloumi cheese samples. Furthermore, milk and cheese that were collected during spring months contained lower saturated and higher unsaturated lipids than the other sampling seasons.

## Figures and Tables

**Table 1:** Effect of farming system (organic vs conventional) and season on the chemical composition of bovine, caprine and ovine milk.

Parameter	Farming System ^1^		Season ^2^					*p*-Value ^3^	
	Con	Org	Aut	Spr	Sum	Win	*SEM* ^4^	F	S
Bovine milk	*n* = 96	*n* = 12	*n* = 27	*n* = 27	*n* = 27	*n* = 27			
Fat, %	3.46	3.40	3.53	3.70	3.43	3.76	0.10	NS	NS
Protein, %	3.76	3.62	3.74	3.75	3.68	3.77	0.05	**	NS
Caprine milk	*n* = 40	*n* = 20	*n* = 15	*n* = 15	*n* = 15	*n* = 15			
Fat, %	4.11	4.14	4.38	4.07	4.03	4.51	0.40	NS	NS
Protein, %	4.03	3.76	3.86 ^b^	3.83 ^b^	3.72 ^b^	4.09 ^a^	0.04	***	**
Ovine milk	*n* = 40	*n* = 12	*n* = 13	*n* = 13	*n* = 13	*n* = 13			
Fat, %	5.34	5.57	5.31	5.17	5.29	5.60	0.20	NS	NS
Protein, %	4.64	4.58	4.66	4.60	4.56	4.66	0.10	NS	NS

^1^ Con = conventional; Org = organic, ^2^ Aut = autumn; Spr = Spring; Sum = Summer; Win = Winter, ^a–b^ Means within a row not sharing a common superscript differ due to season (*p* < 0.05), ^3^ Probability of significant effects due to farming system (F) and season (S), **^4^** Standard Error of the Mean; ** *p* < 0.01; *** *p* < 0.001; NS: Non-significant.

**Table 2:** The effect of farming system (organic vs conventional) and season on the fatty acid composition (expressed as a percentage of total fatty acid methyl esters) of bovine milk.

Parameter	Farming System ^1^		Season ^2^				*SEM* ^4^	*p*-Value ^3^	
	Con	Org	Aut	Spr	Sum	Win		F	S
	*n* = 96	*n* = 12	*n* = 27	*n* = 27	*n* = 27	*n* = 27			
Short- and medium-chain SFA ^5^									
C4:0	2.50	2.67	2.67	2.50	2.64	2.30	0.06	NS	NS
C6:0	1.95	1.93	1.93	1.95	2.12	1.77	0.02	NS	NS
C8:0	1.39	1.31	1.27 ^b^	1.45 ^a^	1.47 ^a^	1.20 ^b^	0.05	NS	**
C10:0	3.15	2.82	2.96 ^b^	3.07 ^b^	3.41 ^a^	2.98 ^b^	0.11	**	**
C12:0	3.83	3.15	3.59	3.70	3.94	3.68	0.10	***	†
C14:0	11.47	10.37	11.29 ^a^	10.52 ^b^	11.69 ^a^	11.70 ^a^	0.09	***	**
Sum	24.31	22.25	23.73	23.24	25.31	23.67	0.44	***	†
Odd- and branched-chain FA ^6^									
C11:0	0.084	0.050	0.078	0.083	0.085	0.066	0.01	***	NS
C13:0	0.144	0.100	0.132	0.151	0.146	0.118	0.01	***	†
iso C14:0	0.14	0.13	0.12 ^b^	0.18 ^a^	0.14 ^b^	0.13 ^b^	0.01	NS	***
C15:0	1.43	1.17	1.37 ^b^	1.57 ^a^	1.38 ^b^	1.23 ^b^	0.07	***	**
ant/iso C15:0	0.68	0.56	0.55 ^b^	0.80 ^a^	0.71 ^a^	0.56 ^b^	0.02	***	***
iso C16:0	0.35	0.29	0.30 ^b^	0.42 ^a^	0.33 ^b^	0.32 ^b^	0.01	**	***
C17:0	0.70	0.66	0.64 ^c^	0.83 ^a^	0.72 ^b^	0.59 ^c^	0.02	NS	***
iso C17:0	0.38	0.36	0.24 ^c^	0.53 ^a^	0.37 ^b^	0.37 ^b^	0.04	NS	***
ant/iso C17:0	0.67	0.55	0.58 ^b^	0.71 ^a^	0.65 ^a,b^	0.66 ^a^	0.02	***	**
Sum	4.49	3.77	3.56 ^c^	5.64 ^a^	4.18 ^b^	4.11 ^b^	0.20	***	***
Long-chain SFA									
C16:0	30.68	26.20	31.55 ^a^	26.63 ^c^	29.54 ^b^	32.36 ^a^	0.45	***	***
C18:0	10.25	12.80	9.82 ^b^	11.99 ^a^	10.26 ^b^	10.47 ^b^	0.25	***	**
C20:0	0.23	0.29	0.14 ^b^	0.47 ^a^	0.19 ^b^	0.18 ^b^	0.01	**	***
Sum	41.12	39.20	41.51 ^b^	38.89 ^c^	39.93 ^c^	43.00 ^a^	0.35	***	***
MUFA ^7^									
C10:1 cis-9	0.33	0.26	0.31 ^b^	0.36 ^a^	0.35 ^a,b^	0.28 ^c^	0.02	***	***
C12:1 cis-9	0.11	0.06	0.11	0.09	0.11	0.10	0.01	***	NS
C14:1 cis-9	1.23	0.96	1.40	1.08	1.26	1.03	0.05	**	†
C16:1 cis-9	1.31	0.98	1.04 ^b^	1.73 ^a^	0.89 ^b,c^	1.37 ^ab^	0.15	*	**
C18:1 cis-9	19.78	23.10	21.23 ^a^	20.34 ^a,b^	19.06 ^b^	20.38 ^ab^	0.30	***	***
C18:1 trans-11	0.70	0.96	0.45 ^b^	1.38 ^a^	0.55 ^b^	0.59 ^b^	0.03	**	***
Other C18:1 ^8^	0.50	0.98	0.61 ^a,b^	0.79 ^a^	0.53 ^b,c^	0.33 ^c^	0.07	***	**
Sum	23.37	26.27	24.67 ^a^	24.70 ^a^	22.41 ^b^	23.44 ^a,b^	0.47	***	**
PUFA ^9^									
C18:2n-6, LA	2.22	3.46	2.50	2.36	2.47	2.30	0.09	***	NS
CLA—cis-9, trans-11	0.34	0.60	0.37 ^b^	0.50 ^a^	0.35 ^b^	0.30 ^b^	0.03	***	***
C18:3n-3, ALA	0.25	0.50	0.26	0.36	0.27	0.25	0.05	***	†
C20:4n-6	0.19	0.20	0.16 ^b^	0.27 ^a^	0.19 ^b^	0.17 ^b^	0.01	NS	***
Sum	3.06	4.76	3.29 ^a,b^	3.65 ^a^	3.37 ^a,b^	3.00 ^b^	0.59	***	***
Calculated values									
LA:ALA	9.83	7.30	9.47	7.57	8.82	8.38	0.90	**	NS
Atherogenic index ^10^	3.09	2.30	2.92 ^a^	2.60 ^b^	3.19 ^a^	3.16 ^a^	0.03	***	*

^1^ Con = conventional; Org = organic, ^2^ Aut = autumn; Spr = Spring; Sum = Summer; Win = Winter; ^3^ Probability of significant effects due to farming system (F) and season (S); **^4^** Standard Error of the Mean; ^a–c^ Means within a row not sharing a common superscript differ due to season (*p* < 0.05); ^5^ Saturated Fatty Acids; ^6^ Fatty Acids; ^7^ Monounsaturated Fatty Acids; ^8^ Other C18:1: C18:1 cis-11, C18:1 cis-12, C18:1 trans-13; ^9^ Polyunsaturated Fatty Acids; ^10^Atherogenic index = (C12:0 + 4 × C14:0 + C16:0)/(ΣMUFA + ΣPUFA); * *p* < 0.05; ** *p* < 0.01; *** *p* < 0.001; NS: Non-significant; † *p* < 0.1: tendency.

**Table 3:** Effect of farming system (organic vs conventional) and season on the fatty acid composition (expressed as a percentage of total fatty acid methyl esters) of caprine milk.

Parameter	Farming System ^1^		Season ^2^				*SEM* ^4^	*p*-Value ^3^	
	Con	Org	Aut	Spr	Sum	Win		F	S
	*n* = 40	*n* = 20	*n* = 15	*n* = 15	*n* = 15	*n* = 15			
Short- and medium-chain SFA ^5^									
C4:0	2.00	1.66	1.87	1.96	1.95	1.61	0.05	***	NS
C6:0	2.25	1.98	2.11	2.23	2.21	1.94	0.10	**	NS
C8:0	2.69	2.49	2.55	2.72	2.60	2.48	0.11	†	NS
C10:0	6.53	7.01	6.45	7.15	7.17	6.17	0.45	NS	NS
C12:0	4.81	4.36	5.23 ^a^	4.14 ^b^	4.34 ^b^	4.75 ^a,b^	0.15	**	***
C14:0	11.02	9.32	11.69 ^a^	9.42 ^c^	9.65 ^b,c^	10.38 ^b^	0.23	***	***
Sum	29.31	26.85	29.86	27.79	27.62	27.03	0.65	**	NS
Odd- and branched-chain FA ^6^									
C11:0	0.14	0.13	0.15	0.14	0.12	0.13	0.01	NS	NS
C13:0	0.11	0.09	0.12	0.11	0.10	0.09	0.01	*	NS
iso C14:0	0.11	0.07	0.11	0.09	0.10	0.07	0.001	***	†
C15:0	1.09	0.81	1.03	1.01	0.95	0.88	0.03	***	†
ant/iso C15:0	0.52	0.30	0.49	0.42	0.45	0.36	0.02	***	†
iso C16:0	0.32	0.21	0.30	0.26	0.29	0.26	0.01	***	NS
C17:0	0.74	0.60	0.60 ^b^	0.79 ^a^	0.66 ^b^	0.66 ^b^	0.02	***	***
iso C17:0	0.50	0.32	0.42 ^a,b^	0.51 ^a^	0.42 ^a,b^	0.36 ^b^	0.01	***	**
ant/iso C17:0	0.62	0.44	0.58	0.54	0.58	0.50	0.01	***	†
Sum	4.20	2.99	3.65	3.87	3.53	3.33	0.15	***	NS
Long-chain SFA									
C16:0	27.38	24.60	28.30 ^a^	23.74 ^c^	25.90 ^b^	27.00 ^a,b^	0.65	***	***
C18:0	10.00	11.17	8.66 ^b^	12.01 ^a^	10.77 ^a^	10.56 ^a^	0.65	**	***
C20:0	0.29	0.22	0.26	0.28	0.27	0.23	0.02	*	NS
Sum	37.62	35.93	37.04	35.73	36.86	37.46	0.41	**	†
MUFA ^7^									
C10:1 cis-9	0.27	0.19	0.31 ^a^	0.22 ^b^	0.21 ^b^	0.21 ^b^	0.02	***	***
C12:1 cis-9	0.065	0.045	0.09 ^a^	0.04 ^b^	0.05 ^b^	0.06 ^b^	0.01	**	***
C14:1 cis-9	0.24	0.15	0.27 ^a^	0.12 ^b^	0.11 ^b^	0.26 ^a^	0.04	***	***
C16:1 cis-9	0.82	0.60	0.90 ^a^	0.72 ^a^	0.44 ^b^	0.82 ^a^	0.04	***	***
C18:1 cis-9	19.30	21.96	19.19 ^b^	20.75 ^a,b^	19.71 ^a,b^	21.81 ^a^	0.56	***	**
C18:1 trans-11	0.64	0.70	0.39 ^b^	1.47 ^a^	0.48 ^b^	0.33 ^b^	0.20	NS	***
Other C18:1 ^8^	0.58	0.60	0.57 ^a,b^	0.79 ^a^	0.58 ^a,b^	0.43 ^b^	0.06	NS	*
Sum	21.38	23.78	21.07 ^c^	23.67 ^a^	21.26 ^b,c^	23.34 ^a,b^	0.65	***	***
PUFA ^9^									
C18:2n-6, LA	2.79	4.54	3.18 ^b,c^	4.15 ^a^	3.88 ^b,a^	3.02 ^c^	0.15	***	***
CLA—cis-9, trans-11	0.42	0.88	0.64	0.70	0.65	0.50	0.04	***	NS
C18:3n-3, ALA	0.32	0.45	0.35 ^b^	0.50 ^a^	0.36 ^b^	0.29 ^b^	0.02	***	***
C20:4n-6	0.213	0.215	0.20	0.25	0.19	0.21	0.01	NS	†
Sum	3.30	5.70	4.17 ^b^	4.86 ^a^	4.18 ^b^	4.02 ^b^	0.37	***	*
Calculated values									
LA:ALA	11.30	10.54	10.50	11.29	11.07	10.37	0.35	†	NS
Atherogenic index ^10^	3.16	2.29	3.27 ^a^	2.42 ^b^	2.71 ^b^	2.77 ^b^	0.23	***	***

^1^ Con = conventional; Org = organic; ^2^ Aut = autumn; Spr = Spring; Sum = Summer; Win = Winter; ^3^ Probability of significant effects due to farming system (F) and season (S); **^4^** Standard Error of the Mean; ^a–c^ Means within a row not sharing a common superscript differ due to season (*p* < 0.05); ^5^ Saturated Fatty Acids; ^6^ Fatty Acids; ^7^ Monounsaturated Fatty Acids; ^8^ Other C18:1: C18:1 cis-11, C18:1 cis-12, C18:1 trans-13; ^9^ Polyunsaturated Fatty Acids; ^10^Atherogenic index = (C12:0 + 4 × C14:0 + C16:0)/(ΣMUFA + ΣPUFA); * *p* < 0.05; ** *p* < 0.01; *** *p* < 0.001; NS: Non-significant; † *p* < 0.1: tendency.

**Table 4:** Effect of farming system (organic vs conventional) and season on the fatty acid composition (expressed as a percentage of total fatty acid methyl esters) of ovine milk.

Parameter	Farming System ^1^		Season ^2^				*SEM* ^4^	*p*-Value ^3^	
	Con	Org	Aut	Spr	Sum	Win		F	S
	*n* = 40	*n* = 12	*n* = 13	*n* = 13	*n* = 13	*n* = 13			
Short- and medium-chain SFA ^5^									
C4:0	2.52	2.73	2.89	2.47	2.54	2.35	0.30	NS	NS
C6:0	2.33	2.34	2.54	2.28	2.30	2.23	0.31	NS	NS
C8:0	2.43	2.37	2.49	2.44	2.31	2.47	0.26	NS	NS
C10:0	5.59	6.12	4.95	5.98	6.01	5.79	0.42	NS	NS
C12:0	4.86	4.40	4.86	4.63	4.46	5.24	0.65	NS	NS
C14:0	11.92	10.80	12.68	10.64	11.57	12.21	2.27	†	†
Sum	29.67	28.78	31.04	27.41	29.45	29.01	1.50	NS	NS
Odd- and branched-chain FA ^6^									
C11:0	0.11	0.09	0.09	0.14	0.10	0.11	0.002	NS	NS
C13:0	0.11	0.10	0.10	0.13	0.11	0.11	0.001	†	NS
C15:0	1.12	0.92	0.97 ^b^	1.20 ^a^	1.19 ^a^	0.99 ^b^	0.02	**	*
C17:0	0.69	0.62	0.60	0.77	0.70	0.69	0.02	NS	NS
Sum	3.96	2.14	3.20	3.37	2.86	2.76	0.22	*	NS
Long-chain SFA									
C16:0	26.80	23.93	27.78	23.48	26.71	27.49	1.01	*	†
C18:0	9.72	10.14	8.59 ^b^	11.52 ^a^	9.49 ^b^	9.47 ^b^	0.45	NS	**
C20:0	0.28	0.31	0.25 ^b,c^	0.34 ^a^	0.32 ^a,b^	0.23 ^c^	0.01	NS	**
Sum	36.80	34.39	34.76	35.18	36.39	36.05	1.00	†	NS
MUFA ^7^									
C10:1 cis-9	0.30	0.29	0.34	0.31	0.29	0.25	0.01	NS	†
C12:1 cis-9	0.06	0.05	0.08 ^a^	0.05 ^b^	0.05 ^b^	0.06 ^a,b^	0.001	NS	*
C14:1 cis-9	0.20	0.21	0.24	0.23	0.14	0.11	0.01	NS	NS
C16:1 cis-9	0.89	1.02	1.15 ^a^	1.02 ^a^	0.61 ^b^	0.91 ^a,b^	0.13	NS	*
C18:1 cis-9	20.64	23.51	21.50	19.85	21.52	21.40	0.84	*	NS
C18:1 trans-11	0.96	1.03	0.68 ^b^	1.79 ^a^	0.71 ^b^	0.86 ^b^	0.09	*	***
Other C18:1 ^8^	0.97	1.16	0.59 ^c^	1.71 ^a^	0.99 ^b^	0.83 ^b,c^	0.08	**	***
Sum	23.20	25.00	24.19	22.80	22.73	23.92	1.69	NS	NS
PUFA ^9^									
C18:2n-6, LA	1.39	1.97	1.72	1.82	1.88	1.28	0.26	*	NS
CLA—cis-9, trans-11	0.52	0.75	0.43 ^b^	0.76 ^a^	0.63 ^a^	0.45 ^b^	0.02	**	*
C18:3n-3, ALA	0.35	0.48	0.31	0.46	0.42	0.31	0.02	*	NS
C20:4n-6	0.23	0.24	0.20	0.28	0.23	0.22	0.004	NS	NS
Sum	2.35	3.59	2.24	2.93	2.75	2.34	0.42	**	†
Calculated values									
LA:ALA	4.06	4.31	4.33	4.84	3.94	3.61	.0.40	NS	NS
Atherogenic index ^10^	3.14	2.33	3.22 ^a^	2.63 ^b^	2.82 ^b^	3.15 ^a^	0.40	*	*

^1^ Con = conventional; Org = organic; ^2^ Aut = autumn; Spr = Spring; Sum = Summer; Win = Winter; ^3^ Probability of significant effects due to farming system (F) and season (S); **^4^** Standard Error of the Mean; ^a–c^ Means within a row not sharing a common superscript differ due to season (*p* < 0.05); ^5^ Saturated Fatty Acids; ^6^ Fatty Acids; ^7^ Monounsaturated Fatty Acids; ^8^ Other C18:1: C18:1 cis-11, C18:1 cis-12, C18:1 trans-13; ^9^ Polyunsaturated Fatty Acids; ^10^Atherogenic index = (C12:0 + 4 × C14:0 + C16:0)/(ΣMUFA + ΣPUFA); * *p* < 0.05; ** *p* < 0.01; *** *p* < 0.001; NS: Non-significant; † *p* < 0.1: tendency.

**Table 5:** Effect of farming system (organic vs conventional) and season on the fatty acid composition (expressed as a percentage of the total fatty acid methyl esters) of Halloumi cheese.

Parameter	Farming System ^1^		Season ^2^				*SEM* ^4^	*p*-Value ^3^	
	Con	Org	Aut	Spr	Sum	Win		F	S
	*n* = 48	*n* = 48	*n* = 24	*n* = 24	*n* = 24	*n* = 24			
Short-chain SFA ^5^									
C5:0	0.039	0.033	0.043 ^a^	0.042 ^a^	0.041 ^a^	0.021 ^b^	0.003	NS	*
C6:0	1.22	1.10	1.13	1.15	1.22	1.19	0.01	***	NS
C8:0	1.34	1.13	1.16	1.29	1.39	1.23	0.04	**	†
C10:0	3.45	2.97	3.17	3.23	3.62	3.06	0.10	**	NS
Medium-chain SFA									
C12:0	3.11	2.71	2.94	2.94	3.03	2.97	0.05	***	NS
C14:0	9.03	8.54	8.98 ^a^	8.66 ^b^	8.93 ^a,b^	8.83 ^a,b^	0.06	***	***
iso C14:0	0.092	0.085	0.087	0.087	0.097	0.086	0.002	*	NS
iso C15:0	0.246	0.224	0.238	0.235	0.246	0.230	0.004	***	NS
ant/iso C15:0	0.89	0.88	0.95	0.85	0.84	0.91	0.04	NS	NS
C16:0	27.86	25.76	27.70 ^a^	25.94 ^b^	27.35 ^a^	27.25 ^a^	0.15	***	***
iso C16:0	0.33	0.30	0.31	0.32	0.34	0.33	0.005	***	NS
C17:0	0.85	0.72	0.74 ^b^	0.84 ^a^	0.81 ^a^	0.83 ^a^	0.01	***	***
iso C17:0	0.67	0.58	0.62	0.63	0.66	0.66	0.01	***	†
Long-chain SFA									
C18:0	12.63	13.95	12.27 ^b^	13.38 ^a^	13.42 ^a^	13.36 ^a^	0.15	***	***
MUFA ^6^									
C10:1 cis-9	0.18	0.15	0.18 ^a^	0.15 ^b^	0.16 ^a,b^	0.17 ^a,b^	0.004	***	***
C16:1 cis-9	1.43	1.30	1.60 ^a^	1.19 ^b^	1.16 ^b^	1.52 ^a,b^	0.07	NS	***
C18:1 cis-9	25.62	26.95	26.31	26.29	25.47	26.50	0.15	***	NS
C18:1 trans-11	0.71	0.83	0.70 ^b^	0.90 ^a^	0.69 ^b^	0.70 ^b^	0.03	*	***
Other C18:1 ^7^	0.99	1.50	1.22	1.21	1.07	1.16	0.03	***	NS
PUFA ^8^									
C18:2n-6, LA	3.47	4.62	3.93 ^b^	4.32 ^a^	3.68 ^c^	3.74 ^b,c^	0.05	***	***
CLA—cis-9, trans-11	0.79	1.07	0.99 ^a^	1.01 ^a^	0.82 ^b^	0.77 ^b^	0.02	***	**
C18:3n-3, ALA	0.61	0.72	0.62 ^b,c^	0.88 ^a^	0.68 ^b^	0.47 ^c^	0.03	**	***
C20:4n-6	0.29	0.30	0.27	0.29	0.28	0.32	0.01	NS	NS
Calculated values									
SFA	62.12	59.28	60.56 ^b,c^	59.84 ^c^	62.52 ^a^	61.32 ^b^	0.15	***	***
MUFA	28.93	30.63	30.03 ^a^	29.58 ^a^	28.59 ^b^	30.02 ^a^	0.15	***	*
PUFA	5.01	6.69	5.79 ^a,b^	6.27 ^a^	5.31 ^b^	5.25 ^b^	0.12	***	***
LA:ALA	7.03	6.70	6.73	5.79	6.29	8.65	0.52	NS	NS
Atherogenic index ^9^	1.98	1.68	1.87 ^b^	1.77 ^c^	1.96 ^a^	1.86 ^b^	0.01	***	***

^1^ Con = conventional; Org = organic; ^2^ Aut = autumn; Spr = Spring; Sum = Summer; Win = Winter; ^3^ Probability of significant effects due to farming system (F) and season (S); **^4^** Standard Error of the Mean; ^a–c^ Means within a row not sharing a common superscript differ due to season (*p* < 0.05); ^5^ Saturated Fatty Acids; ^6^ Monounsaturated Fatty Acids; ^7^ Other C18:1: C18:1 cis-11, C18:1 cis-12, C18:1 trans-13; ^8^ Polyunsaturated Fatty Acids; ^9^Atherogenic index = (C12:0 + 4 × C14:0 + C16:0)/(ΣMUFA + ΣPUFA); ** *p* < 0.01; *** *p* < 0.001; NS: Non-significant; † *p* < 0.1: tendency.

## Data Availability

The raw data presented in this study are available on request from the corresponding author.

## References

[1] Chilliard Y., Glasser F., Ferlay A., Bernard L., Rouel J., Doreau M. (2007). Diet, rumen biohydrogenation and nutritional quality of cow and goat milk fat. Eur. J. Lipid Sci. Technol..

[2] Jensen R.G. (2002). The Composition of Bovine Milk Lipids: January 1995 to December 2000. J. Dairy Sci..

[3] Walker G.P., Dunshea F.R., Doyle P.T. (2004). Effects of nutrition and management on the production and composition of milk fat and protein: A review. Aust. J. Agric. Res..

[4] Collomb M., Schmid A., Sieber R., Wechsler D., Ryhänen E.L. (2006). Conjugated linoleic acids in milk fat: Variation and physiological effects. Int. Dairy J..

[5] Dewhurst R.J., Shingfield K.J., Lee M.R.F., Scollan N.D. (2006). Increasing the concentrations of beneficial polyunsaturated fatty acids in milk produced by dairy cows in high-forage systems. Anim. Feed Sci. Technol..

[6] Davis H., Chatzidimitriou E., Leifert C., Butler G. (2020). Evidence that forage-fed cows can enhance milk quality. Sustainability.

[7] Tzamaloukas O., Neofytou M.C., Simitzis P.E. (2021). Application of Olive By-Products in Livestock with Emphasis on Small Ruminants: Implications on Rumen Function, Growth Performance, Milk and Meat Quality. Animals.

[8] Popa M.E., Mitelut A.C., Popa E.E., Stan A., Popa V.I. (2019). Organic foods contribution to nutritional quality and value. Trends Food Sci. Technol..

[9] Ferlay A., Bernard L., Meynadier A., Malpuech-Brugère C. (2017). Production of trans and conjugated fatty acids in dairy ruminants and their putative effects on human health: A review. Biochimie.

[10] Adler S.A., Jensen S.K., Govasmark E., Steinshamn H. (2013). Effect of short-term versus long-term grassland management and seasonal variation in organic and conventional dairy farming on the composition of bulk tank milk. J. Dairy Sci..

[11] Butler G., Nielsen J., Slots T., Seal C., Eyre M., Sanderson R., Leifert C. (2008). Fatty acid and fat-soluble antioxidant concentrations in milk from high- and low-input conventional and organic systems: Seasonal variation. J. Sci. Food Agric..

[12] Butler G., Stergiadis S., Seal C., Eyre M., Leifert C. (2011). Fat composition of organic and conventional retail milk in northeast England. J. Dairy Sci..

[13] Ellis K.A., Innocent G., Grove-White D., Cripps P., McLean W.G., Howard C.V., Mihm M. (2006). Comparing the fatty acid composition of organic and conventional milk. J. Dairy Sci..

[14] Liu N., Pustjens A.M., Erasmus S.W., Yang Y., Hettinga K., van Ruth S.M. (2020). Dairy farming system markers: The correlation of forage and milk fatty acid profiles from organic, pasture and conventional systems in the Netherlands. Food Chem..

[15] Tudisco R., Cutrignelli M.I., Calabrò S., Piccolo G., Bovera F., Guglielmelli A., Moniello G., Infascelli F. (2010). Influence of organic systems on milk fatty acid profile and CLA in goats. Small Rumin. Res..

[16] Palupi E., Jayanegara A., Ploeger A., Kahl J. (2012). Comparison of nutritional quality between conventional and organic dairy products: A meta-analysis. J. Sci. Food Agric..

[17] Srednicka-Tober D., Barański M., Seal C.J., Sanderson R., Benbrook C., Steinshamn H., Gromadzka-Ostrowska J., Rembiałkowska E., Skwarło-Sońta K., Eyre M. (2016). Higher PUFA and n-3 PUFA, conjugated linoleic acid, α-tocopherol and iron, but lower iodine and selenium concentrations in organic milk: A systematic literature review and meta- and redundancy analyses. Br. J. Nutr..

[18] Givens D.I., Lovegrove J.A. (2016). Higher PUFA and n-3 PUFA, conjugated linoleic acid, α-tocopherol and iron, but lower iodine and selenium concentrations in organic milk: A systematic literature review and meta-and redundancy analyses. Br. J. Nutr..

[19] Collomb M., Bisig W., Bütikofer U., Sieber R., Bregy M., Etter L. (2008). Fatty acid composition of mountain milk from Switzerland: Comparison of organic and integrated farming systems. Int. Dairy J..

[20] Slots T., Sorensen J., Nielsen J. (2008). Tocopherol, carotenoids and fatty acid composition in organic and conventional milk. Milchwissenschaft.

[21] Massouras T., Maragoudakis S., Hadjigeorgiou I. (2018). Differences in sheep milk characteristics focusing on fatty acid profile between conventional and organic farming system. Arch. Dairy Res. Technol..

[22] Tsiplakou E., Kotrotsios V., Hadjigeorgiou I., Zervas G. (2010). Differences in sheep and goats milk fatty acid profile between conventional and organic farming systems. J. Dairy Res..

[23] Schwendel B.H., Wester T.J., Morel P.C.H., Tavendale M.H., Deadman C., Shadbolt N.M., Otter D.E. (2015). Invited review: Organic and conventionally produced milk-An evaluation of factors influencing milk composition. J. Dairy Sci..

[24] Tsiafoulis C.G., Papaemmanouil C., Alivertis D., Tzamaloukas O., Miltiadou D., Balayssac S., Malet-Martino M., Gerothanassis I.P. (2019). NMR-based metabolomics of the lipid fraction of organic and conventional bovine milk. Molecules.

[25] AOAC (Association of Official Agricultural Chemists) (2005). Official Methods of Analysis.

[26] Papademas P., Tamime A.Y. (2006). Halloumi cheese. Brined Cheeses.

[27] Tzamaloukas O., Orford M., Miltiadou D., Papachristoforou C. (2015). Partial suckling of lambs reduced the linoleic and conjugated linoleic acid contents of marketable milk in Chios ewes. J. Dairy Sci..

[28] Neofytou M.C., Miltiadou D., Sfakianaki E., Constantinou C., Symeou S., Sparaggis D., Hager-Theodorides A.L., Tzamaloukas O. (2020). The use of ensiled olive cake in the diets of Friesian cows increases beneficial fatty acids in milk and Halloumi cheese and alters the expression of SREBF1 in adipose tissue. J. Dairy Sci..

[29] ISO (International Organization for Standardization) (2000). Animal and Vegetable Fats and Oils—Preparation of Methyl Esters of Fatty Acids.

[30] Kusche D., Kuhnt K., Ruebesam K., Rohrer C., Nierop A.F., Jahreis G., Baars T. (2014). Fatty acid profiles and antioxidants of organic and conventional milk from low- and high-input systems during outdoor period. J. Sci. Food Agric..

[31] Stergiadis S., Leifert C., Seal C.J., Eyre M.D., Nielsen J.H., Larsen M.K., Slots T., Steinshamn H., Butler G. (2012). Effect of feeding intensity and milking system on nutritionally relevant milk components in dairy farming systems in the north east of England. J. Agric. Food Chem..

[32] Lopez A., Vasconi M., Moretti V.M., Bellagamba F. (2019). Fatty Acid Profile in Goat Milk from High- and Low-Input Conventional and Organic Systems. Animals.

[33] Kuczyńska B., Puppel K., Gołȩbiewski M., Metera E., Sakowski T., Słoniewski K. (2012). Differences in whey protein content between cow’s milk collected in late pasture and early indoor feeding season from conventional and organic farms in Poland. J. Sci. Food Agric..

[34] Malissiova E., Tzora A., Katsioulis A., Hatzinikou M., Tsakalof A., Arvanitoyannis I.S., Govaris A., Hadjichristodoulou C. (2015). Relationship between production conditions and milk gross composition in ewe’s and goat’s organic and conventional farms in central Greece. Dairy Sci. Technol..

[35] Stergiadis S., Berlitz C.B., Hunt B., Garg S., Givens D.I., Kliem K.E. (2019). An update to the fatty acid profiles of bovine retail milk in the United Kingdom: Implications for nutrition in different age and gender groups. Food Chem..

[36] Butler G., Stergiadis S., Givens I.D. (2020). Organic milk: Does it confer health benefits?. Milk and Dairy Foods—Their Functionality in Human Health and Disease.

[37] Rego O.A.D., Regalo S.M.M., Rosa H.J.D., Alves S.P., Borba A.E.S.D., Bessa R.J.B., Cabrita A.R.J., Fonseca A.J.M. (2008). Effects of grass silage and soybean meal supplementation on milk production and milk fatty acid profiles of grazing dairy cows. J. Dairy Sci..

[38] Shingfield K.J., Reynolds C.K., Lupoli B., Toivonen V., Yurawecz M.P., Delmonte P., Griinari J.M., Grandison A.S., Beever D.E. (2005). Effect of forage type and proportion of concentrate in the diet on milk fatty acid composition in cows given sunflower oil and fish oil. Anim. Sci..

[39] Butler G., Stergiadis S., Chatzidimitriou E., Franceschin E., Davis H.R., Leifert C., Steinshamn H. (2019). Differing responses in milk composition from introducing rapeseed and naked oats to conventional and organic dairy diets. Sci. Rep..

[40] Benbrook C.M., Butler G., Latif M.A., Leifert C., Davis D.R. (2013). Organic production enhances milk nutritional quality by shifting fatty acid composition: A United States-wide, 18-month study. PLoS ONE.

[41] Capuano E., Gravink R., Boerrigter-Eenling R., van Rut S.M. (2015). Fatty acid and triglycerides profiling of retail organic, conventional and pasture milk: Implications for health and authenticity. Int. Dairy J..

[42] Schwendel B.H., Wester T.J., Morel P.C., Fong B., Tavendale M.H., Deadman C., Shadbolt N.M., Otter D.E. (2017). Pasture feeding conventional cows removes differences between organic and conventionally produced milk. Food Chem..

[43] Heck J.M.L., van valenberg H.J.F., Dijkstra J., van Hooijdonk A.C.M. (2009). Seasonal variation in the Dutch bovine raw milk composition. J. Dairy Sci..

[44] Lock A.L., Garnsworthy P.C. (2003). Seasonal variation in milk conjugated linoleic acid and δ9-desaturase activity in dairy cows. Livest. Prod. Sci..

[45] Tsiplakou E., Mountzouris K.C., Zervas G. (2006). Concentration of conjugated linoleic acid in grazing sheep and goat milk fat. Livest. Sci..

[46] Altomonte I., Conte G., Serra A., Mele M., Cannizzo L., Salari F., Martini M. (2019). Nutritional characteristics and volatile components of sheep milk products during two grazing seasons. Small Rum. Res..

[47] Salari F., Altomonte I., Ribeiro N.L., Ribeiro M.N., Bozzi R., Martini M. (2016). Effects of season on the quality of Garfagnina goat milk. Ital. J. Anim. Sci..

[48] Rutkowska J., Adamska A., Bialek M. (2012). Fatty acid profile of the milk of cows reared in the mountain region of Poland. J. Dairy Res..

[49] Ferlay A., Martin B., Pradel P., Coulon J.B., Chilliard Y. (2006). Influence of grass-based diets on milk fatty acid composition and milk lipolytic system in Tarentaise and Montbéliarde cow breeds. J. Dairy Sci..

[50] Prandini A., Sigolo S., Piva G. (2009). Conjugated linoleic acid (CLA) and fatty acid composition of milk, curd and Grana Padano cheese in conventional and organic farming systems. J. Dairy Res..

[51] Bergamo P., Fedele E., Iannibelli L., Marzillo G. (2003). Fat-soluble vitamin contents and fatty acid composition in organic and conventional Italian dairy products. Food Chem..

[52] Esposito G., Masucci F., Napolitano F., Braghieri A., Romano R., Manzo N., Di Francia A. (2014). Fatty acid and sensory profiles of Caciocavallo cheese as affected by management system. J. Dairy Sci..

[53] Revello Chion A., Tabacco E., Giaccone D., Peiretti P.G., Battelli G., Borreani G. (2010). Variation of fatty acid and terpene profiles in mountain milk and “Toma piemontese” cheese as affected by diet composition in different seasons. Food Chem..

[54] Symeou S., Miltiadou D., Constantinou C., Papademas P., Tzamaloukas O. (2021). Feeding olive cake silage up to 20% of DM intake in sheep improves lipid quality and health-related indices of milk and ovine halloumi cheese. Trop. Anim. Health Prod..

